# Lymphangioleiomyomatosis: Searching for potential biomarkers

**DOI:** 10.3389/fmed.2023.1079317

**Published:** 2023-02-02

**Authors:** Eva Revilla-López, Victoria Ruiz de Miguel, Manuel López-Meseguer, Cristina Berastegui, Meritxell Boada-Pérez, Alberto Mendoza-Valderrey, Marta Arjona-Peris, Marta Zapata-Ortega, Victor Monforte, Carlos Bravo, Antonio Roman, Susana Gómez-Ollés, Berta Sáez-Giménez

**Affiliations:** ^1^Lung Transplant Program, Department of Pulmonology, Hospital Universitari Vall d’Hebron, Barcelona, Spain; ^2^Department of Medicine, Universitat Autònoma de Barcelona, Barcelona, Spain; ^3^Department of Pulmonology, Vall d’Hebron Institut de Recerca, Barcelona, Spain; ^4^CIBER de Enfermedades Respiratorias, Instituto de Salud Carlos III, Madrid, Spain; ^5^Department of Cell Biology, Physiology and Immunology, Universitat Autònoma de Barcelona, Barcelona, Spain

**Keywords:** lymphangioleiomyomatosis, biomarkers, serum, metalloproteinases, VEGF-D, diagnose

## Abstract

**Background:**

Vascular endothelial growth factor-D (VEGF-D) is the most commonly used biomarker for diagnosing lymphangioleiomyomatosis (LAM). However, lung biopsy is often necessary as well; therefore, defining new biomarkers for LAM is crucial. The aim of this study was to describe the diagnostic accuracy of a variety of biomarkers.

**Methods:**

We assessed 13 analytes in serum related to extracellular matrix remodeling, lymphatic involvement and angiogenesis in a cohort of patients with LAM, comparing them with patients with other cystic lung diseases (OCLD) and healthy women. A scoring method based on the cut-point of each VEGF-D and metalloproteinase-2 (MMP-2) was used to evaluate the diagnostic performance of the marker combination.

**Results:**

A total of 97 subjects were recruited: 59 (61%) LAM patients, 18 (19%) OCLD patients, and 20 (20%) healthy female controls. MMP-2 was the only extracellular matrix remodeling biomarker able to differentiate LAM patients from OCLD and healthy patients. Serum MMP-2 was higher in LAM patients [median 578 (465–832) ng/ml] than in patients with OCLD and healthy controls [medians 360 (314–546) and 427 (365–513) ng/ml, respectively (*p* < 0.0001)]. The area under ROC curve (AUC) of MMP-2 was 0.785 and that of VEGF-D 0.815 (*p* = 0.6214). The sensitivity/specificity profiles of each biomarker (54/92% for MMP-2, 59/95% for VEGF-D) yielded a composite score (−6.36 + 0.0059 × VEGF-D + 0.0069 × MMP-2) with higher accuracy than each component alone (AUC 0.88 and sensitivity/specificity 79/87%).

**Conclusion:**

Combining MMP-2 and VEGF-D may increase diagnostic accuracy for LAM.

## 1. Introduction

Lymphangioleiomyomatosis (LAM) is a rare multisystem disease that predominantly affects women and is characterized by the proliferation of abnormal smooth muscle-like cells named LAM cells (1). LAM is secondary to mutations in the tuberous sclerosis complex (TSC) genes, mainly TSC2, which cause the activation of the mammalian target of rapamycin (mTOR) complex (2). LAM cells proliferate in the lungs and the axial lymphatic system, but also invade the airways, pulmonary artery, diaphragm, aorta, and retroperitoneal fat tissue (3, 4).

Serum levels of vascular endothelial growth factor-D (VEGF-D), a ligand for the lymphatic growth-factor receptor VEGFR-3/Flt-4, are higher in most LAM patients than in healthy controls or in patients with other cystic lung diseases (OCLD) (5, 6). It has been suggested that a VEGF-D level of > 800 pg/ml and the presence of pulmonary cysts is diagnostic for LAM (7). However, a VEGF-D level of < 800 pg/ml does not rule out the diagnosis (6). A wide range of VEGF-D levels has been described (8); they are higher in patients with lymphatic disease who develop chylous pleural effusion or ascites than in those without lymphatic involvement, which may explain these findings (9), although the variability may also be due to the differences in the assay kits used and in the access to standardized tests (10). Even though the diagnostic value of VEGF-D is well established, its clinical utility to monitor follow-up and treatment response is not clear (11). Therefore, the search for new LAM biomarkers continues.

In addition to lymphatic involvement, extracellular matrix remodeling, and angiogenesis in LAM (12), other biomarkers may be related to these biological processes. Matrix metalloproteinases (MMP) are a family of endopeptidases involved not only in extracellular matrix degradation but also in cell proliferation, differentiation, cell signaling, and migration (13). Associations between MMP and destructive lesions caused by LAM have been demonstrated (14) and elevated MMP-2, MMP-7, and MMP-9 levels have been observed in serum of LAM patients (15–17). However, these studies only compared LAM patients with healthy controls.

Angiopoietin-1, endostatin, fibroblast growth factor-acidic (FGF-acidic), phosphatidylinositol-glycan biosynthesis class F protein (PIGF), thrombospondin-2 and platelet derived growth factor-AA protein (PDGF-AA) are biomarkers associated with angiogenesis. Little information is available on these biomarkers in LAM; only one study has reported higher levels of endostatin with LAM associated with TSC than in patients with sporadic LAM (S-LAM) and healthy volunteers (18). Von Willebrand factor (vWF) is a protein associated with angiogenesis (19) and is reduced in LAM patients compared to healthy controls (20). Cancer antigen-125 (CA-125) and Krebs von den Lungen 6 (KL-6) are also related to LAM: CA-125 levels have been associated with pleural effusions and reduced pulmonary function (21) and KL-6 levels are higher than in other lung diseases (22).

Thus, the objective of the present study was to describe the diagnostic accuracy of a range of biomarkers in a cohort of LAM patients compared to patients with other cystic diseases and healthy volunteers.

## 2. Materials and methods

### 2.1. Subjects and clinical data

A cross-sectional study was conducted from July 2019 to June 2020. The study population comprised LAM patients with or without lung transplantation (LT), patients with OCLD and healthy females. The cohort of OCLD included Langerhans cell histiocytosis, emphysema, and Birt–Hogg–Dubé syndrome. LAM patients were diagnosed by lung biopsy or by the presence of lung cysts plus: renal angiomyolipomas, tuberous sclerosis, or chylous effusions.

Clinical findings, diagnostic methods, sirolimus treatment, childbirth, menopausal status, lung function, LT, presence of tuberous sclerosis, and extrapulmonary involvement were retrospectively recorded from electronic clinical records.

Cystic lung involvement was graded according to thoracic CT scans in LAM patients and in OCLD patients. Lungs were divided into three equal zones (upper, middle, and lower). Based on the lung volume replaced by cysts within each zone, cyst involvement was graded individually and subjectively as follows: mild disease, less than 30%; moderate disease, cysts involve 30–60%; severe disease, cysts involve greater than 60%, as previously reported (23). All CT scans were retrospectively reviewed by the same operator.

Analysis of sex hormones such as estradiol, luteinizing hormone, follitropin, progesterone, and prolactin, as well as pulmonary function tests (PFTs) were performed at the time of inclusion in the study. PFT followed the American Thoracic Society (ATS)/European Respiratory Society protocols (24).

Sirolimus was indicated in LAM patients in the case of FEV1 < 80%, an angiomyolipoma larger than 4 cm in diameter or multiple angiomyolipomas. In LAM patients who underwent LT, sirolimus was prescribed in the case of chronic lung allograft dysfunction (CLAD) or angiomyolipomas. Sirolimus was given at a dose between 1 and 4 mg once daily adjusted to obtain target trough blood levels between 5 and 15 ng/ml measured with liquid chromatography-mass spectrometry.

The Ethics Committee of the Vall d’Hebron University Hospital (Barcelona, Spain ARB-SIR-2018-01) approved the study and written informed consent was obtained from all subjects.

### 2.2. Blood collection and storage

Blood samples were collected in one BD Vacutainer^®^ Plastic SST II Advance Tube, one BD Vacutainer^®^ Citrate Tube, and one BD Vacutainer^®^ spray-coated K2EDTA tube. The serum tube was allowed to clot at room temperature for 60 min prior to centrifugation at 1,300 *g* at 4°C for 10 min. Serum was obtained and stored at −80°C until used. The EDTA tube was immediately centrifuged after the blood extraction for 15 min at 1,000 *g* at 4°C. Plasma was aliquoted into 1 ml aliquots, and then an additional centrifugation was performed at 10,000 *g* at 4°C for 10 min to obtain platelet-free plasma. All plasma samples were stored at −70°C until used.

### 2.3. MMP-2, MMP-9, endostatin, FGF acidic, PIGF, thrombospondin-2, VEGF-D, VEGF- R3, angiopoietin-1, PDGF-AA, vWF, KL-6, and CA-125 assays

Serum MMP-2 and MMP-9 were measured using Luminex Human Magnetic Assay LXSAHM-02 (R&D Systems, Minneapolis, MN, USA). vWF activity assay was performed on ACLTOP 700 LAS using reagents from the same supplier (IL Werfen, Barcelona, Spain). Serum CA-125 was measured by chemiluminescence assay using Atellica^®^ automated analyzer with reagents from the same supplier (Siemens Healthcare Diagnostics, Erlangen, Germany). Endostatin, FGF acidic, PIGF, thrombospondin-2, angiopoietin-1, and PDGF-AA were measured using Luminex Performance Human Angiogenesis Magnetic Panel A (6-Plex), R&D Systems. VEFD-R3 was measured using Luminex Human Magnetic Assay (1-Plex) LXSAHM-01, R&D Systems. VEGF-D was measured by a human VEGF-D Quantikine^®^ ELISA kit, R&D Systems. KL-6 was measured using a chemiluminescence enzyme immunoassay (CLEIA) by Lumipulse G KL-6 IRC (Fujirebio, Tokyo, Japan).

### 2.4. Statistical analysis

Continuous variables are expressed as means ± standard deviation (SD) and as medians and interquartile range (IQR). Categorical variables are expressed as numbers of cases and proportions. Normality was evaluated in continuous variables using the Shapiro–Wilk test and comparisons of groups were made using analysis of variance (ANOVA). Categorical variables were compared using the chi-square test or Fisher’s exact test, as appropriate. For comparisons between patients the Kruskal–Wallis test was used, as appropriate. A receiver operating characteristic (ROC) curve was created to detect the cut-off point to establish the optimal sensitivity and specificity of each biomarker. The previously reported VEGF-D cut-off value of 800 pg/ml was also analysed. A logistic regression model including VEGF-D and MMP-2 was used to create a composite score. The DeLong test was used to compare ROC curves. A *p*-value < 0.05 was considered statistically significant. SPSS 27.0 software version (IBM SPSS Statistics, Chicago, IL, USA) and GrapPhad Prism 9.1.2 (Graph Pad Software, San Diego, CA, USA) were used for the analysis.

## 3. Results

**FIGURE 1:**
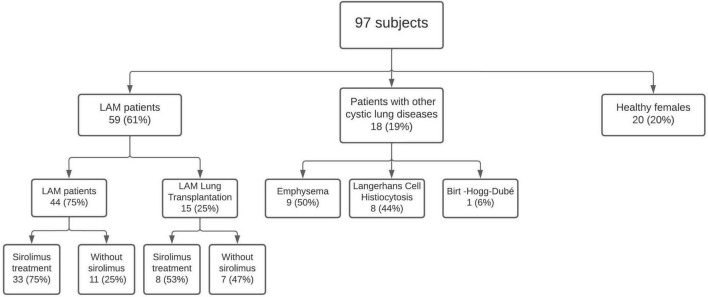
Subjects enrolled in the study.

A total of 97 subjects were recruited: 59 (61%) LAM patients, 18 (19%) OCLD patients, and 20 (20%) healthy female controls. Median time from LAM diagnosis to blood sample collection was 9 (2–17) years. A total of 15 (25%) of the LAM patients had undergone LT prior to recruitment; median time from LT to blood sample collection was 12 (8–13) years. A total of 10 (17%) patients had LAM associated with TSC (TSC-LAM): 2 of them (20%) underwent LT and 6 (75%) were under sirolimus treatment. A total of 9 (50%) of the OCLD patients had emphysema, 8 (44%) had Langerhans cell histiocytosis and 1 (6%) had Birt–Hogg–Dubé syndrome (Figure 1). The method of LAM diagnosis was lung biopsy in 46 (78%) patients, and clinical and radiological findings in 13 (22%).

Regarding cystic lung involvement showed in CT scans: 8 (18%) LAM patients had mild disease, 14 (32%) LAM patients had moderate disease, and 22 (50%) LAM patients had severe disease. As for OCLD patients: 4 (22%) had mild disease, 6 (33%) had moderate disease, and 8 (44%) had severe disease. Dyspnea was the most common initial symptom, recorded in 24 (41%) LAM patients, followed by pneumothorax in 21 (36%), chylothorax in 5 (9%), hemoptysis in 1 (2%), and hematuria also in 1 (2%). A total of 7 (12%) diagnoses were incidental in asymptomatic patients. A total of 28 (47%) patients had renal angiomyolipomas, 12 (20%) of those were bilateral and median size was 25 (14–42) mm × 24 (13–25) mm. A total of 12 (20%) patients had lymphangioleiomyomas. During follow-up, 24 (41%) LAM patients presented recurrent pneumothorax and 5 (9%) developed recurrent chylothorax. No multifocal micronodular pneumocyte hyperplasia was observed on any thoracic CT. Concerning LT, 11 (73%) of them were bilateral and 4 (27%) were single. A total of 11 (73%) LT patients had extrapulmonary lesions: 8 (53%) had angiomyolipomas and 3 (20%) had lymphangioleiomyomas. Only one patient had relapse of LAM after LT and 7 (47%) of them developed CLAD.

A total of 41 (69%) LAM patients were treated with sirolimus: 33 (80%) non-LT patients and 8 (20%) LT patients, with a mean time under sirolimus treatment of 6.9 ± 2.7 and 7.5 ± 2.7 years, respectively. Mean sirolimus dose was 2 ± 0.7 mg/day and mean blood trough levels were 7.6 ± 4.3 ng/ml. Side effects were recorded in 11 (27%) patients and tended to occur early after starting treatment. The most common side effect was the appearance of oral ulcers, recorded in 6 (15%) patients, severe increases in serum cholesterol in 2 (5%), liver enzyme abnormalities in 1 (2%), leg swelling in 1 (2%), and acne in 1 (2%). A total of 3 (5%) LAM patients started sirolimus treatment after the collection of the blood sample for the study. Only 3 (30%) TSC-LAM patients took antiepileptic drugs and 15 (34%) LAM patients were on bronchodilators, with no differences between TSC-LAM and S-LAM (*p* = 0.7). The proportion of patients under other treatments were also comparable between the two groups (Supplemental Table 1).

Table 1 shows the main demographic and clinical characteristics of the study population. There were no differences between groups in post-menopause status, pregnancy, or body mass index, but differences were found in hypertension, hypercholesterolemia, and renal failure between the group of transplanted patients and the rest. In the PFTs, there were only differences in force vital capacity (FVC) as percentage of predicted value.

**TABLE 1:** Demographic and clinical characteristics of the study population.

	LAM (including LT) *n* = 59	LAM (no LT) *n* = 44	LT (*n* = 15)	OCLD (*n* = 18)	Healthy controls (*n* = 20)	*p*-value
Age (years), median (IQR)	52 (46–62)	51 (44–59)	61 (52–66)	57 (46–60)	51 (47–56)	0.037
Post-menopause [*n* (%)]	41 (69)	29 (66)	12 (80)	13 (72)	10 (50)	0.381
Pregnancy [*n* (%)]	37 (63)	26 (59)	11 (73)	14 (78)	10 (50)	0.392
Ex-smokers [*n* (%)]	21 (36)	17 (39)	4 (27)	11 (61)	3 (15)	<0.001
Smokers [*n* (%)]	3 (5)	3 (7)	0 (0)	5 (28)	4 (20)	
BMI (kg/m^2^), mean ± SD	24 ± 4.8	23.6 ± 4	25.2 ± 6.4	24 ± 4.1	23 ± 3.6	0.9214
Hypertension [*n* (%)]	19 (32)	11 (25)	8 (53)	2 (11)	1 (5)	<0.001
Hypercholesterolemia [*n* (%)]	30 (51)	19 (43)	11 (73)	0 (0)	6 (30)	<0.001
Diabetes mellitus [*n* (%)]	7 (12)	4 (9)	3 (20)	1 (6)	0 (0)	0.320
Renal failure (GFR < 60) [*n* (%)]	13 (22)	1 (2)	12 (80)	0 (0)	0 (0)	<0.001
**PFT (mean ± SD)**						
FVC (ml)	2,733 ± 847	2,927 ± 825	2,165 ± 647	2,370 ± 978	–	0.012
FVC (% pred.)	86 ± 21	91 ± 20	72 ± 18	72 ± 26		0.009
FEV1 (ml)	1,850 ± 698	1,922 ± 728	1,638 ± 573	1,629 ± 1,141		0.257
FEV1 (% pred.)	71 ± 22	72 ± 22	69 ± 22	59 ± 35		0.298
FEV1/FVC (%)	68 ± 14	65 ± 15	75 ± 9	61 ± 17		0.059
DLCO (%)	55 ± 25	54 ± 26	68 ± 20	48 ± 30		0.337
Supplemental oxygen [*n* (%)]	6 (10)	5 (11)	1 (6)	7 (39)	–	0.012

LT, lung transplantation; OCLD, other cystic lung diseases; IQR, interquartile range; SD, standard deviation; BMI, body mass index; GFR, glomerular filtration rate (ml/min/1.73 m^2^); PFT, pulmonary function test.

### 3.1. Distribution of serum biomarkers between groups

**FIGURE 2:**
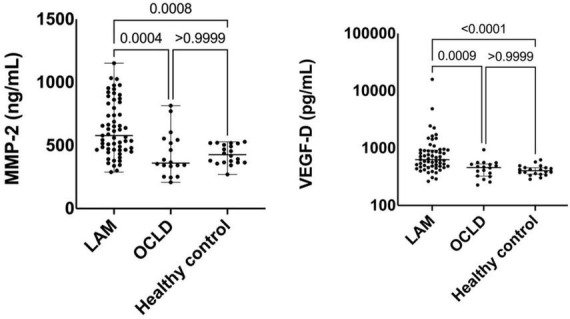
Comparison of serum metalloproteinase-2 (MMP-2) and vascular endothelial growth factor-D (VEGF-D) levels between lymphangioleiomyomatosis (LAM) patients, other cystic lung diseases (OCLD), and healthy controls. Solid lines indicate median and interquartile range (IQR).

Serum MMP-2 was highest in LAM patients, with a median of 578 (465–832) ng/ml compared with medians of 360 (314–546) ng/ml for OCLD and 427 (365–513) ng/ml for healthy controls (*p* < 0.0001) (Figure 2). MMP-2 was able to differentiate between LAM and OCLD patients (*p* = 0.0004). Differences in MMP-2 levels between S-LAM and TSC-LAM were non-significant: median values were 573 (289–1,151) and 502 (404–688) ng/ml, respectively (*p* = 0.181). There were no differences in the LAM group regarding MMP-2 levels and LT: LAM patients had a median of 551 (462–783) ng/ml compared with one of 630 (491–901) ng/ml in LAM LT patients (*p* > 0.999). There were no significant differences either in the LAM group regarding MMP-2 levels and sirolimus treatment: LAM with sirolimus treatment had a median of 578 (466–847) ng/ml, while LAM without sirolimus treatment had a median of 480 (379–656) ng/ml (*p* = 0.081). The difference in MPP-2 levels between healthy controls and LAM patients under sirolimus remained significant (*p* = 0.003). Also, the levels of MMP-2 were comparable among patients with and without bronchodilators: median value was 625 (404–843) and 543 (465–738) ng/ml, respectively (*p* = 0.98).

Serum VEGF-D level was higher in LAM with a median of 629 (482–924) pg/ml, than in OCLD [median 459 (325–530) pg/ml], and healthy controls [median 404 (352–456) pg/ml] (Figure 2). Levels of VEGF-D were higher in TSC-LAM patients [median 733 (523–928) pg/ml] compared with S-LAM [median 554 (457–790) pg/ml] (*p* = 0.039). Considering all LAM patients irrespective of LT, those under sirolimus treatment had a median VEGF-D of 610 (486–886) pg/ml compared to 658 (452–1,638) pg/ml in those without treatment (*p* = 0.457). The difference in VEGF-D levels between healthy controls and LAM patients under sirolimus remained significant (*p* = 0.004). Patients without bronchodilators showed higher VEGF-D levels [median 709 (521–893) pg/ml] compared to those under treatment [median 458 (400–548) pg/ml] (*p* = 0.004).

**FIGURE 3:**
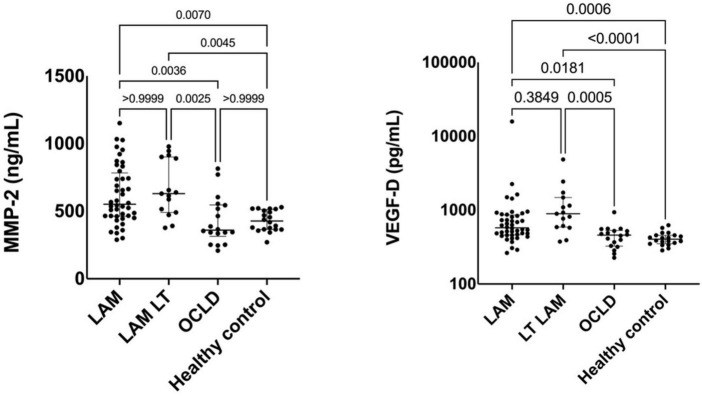
Comparison of serum metalloproteinase-2 (MMP-2) and vascular endothelial growth factor-D (VEGF-D) levels between lymphangioleiomyomatosis (LAM) patients, LAM lung transplantation (LT) patients, other cystic lung diseases (OCLD), and healthy controls. Solid lines indicate median and interquartile range (IQR).

Of note, VEGF-D was persistently elevated in LAM patients after LT though the difference with regard to those without LT was not significant (Figure 3). Median VEGF-D was 577 (461–861) pg/ml in LT-free LAM vs. 893 (589–1,482) pg/ml in the LAM LT group (*p* = 0.384).

Endostatin, PIGF, thrombospondin-2, VEGF-R3, angiopoietin-1, PDGF-AA, MMP-9, CA-125, vWF, FGF-acidic, and KL-6 biomarkers were not able to discriminate between LAM patients and OCLD patients or healthy volunteers (Table 2 and Supplementary Figures 1, 2).

**TABLE 2:** Biomarker levels from LAM patients, QQLT LAM patients, OCLD and healthy controls.

	LAM patients (*n* = 44)	LT LAM (*n* = 15)	OCLD (*n* = 18)	Healthy controls (*n* = 20)	*p*-value
Angiopoietin-1 (pg/ml), median (IQR)	7,178 (5,033–13,450)	9,405 (5,911–11,662)	7,603 (4,726–12,581)	3,766 (2,998–6,604)	0.0035
CA-125 (U/ml), median (IQR)	15 (1.7–25.2)	18.5 (12.6–45.3)	8.2 (6.3–14.5)	10.4 (7.5–12.9)	0.0118
Endostatin (ng/ml), median (IQR)	37 (28–51)	72 (55–80)	35 (26–45)	31 (23–39)	<0.0001
FGF acidic (pg/ml), median (IQR)	16 (12.4–19.2)	16 (11.4–19.2)	16.3 (11.34–18.9)	11.6 (10.3–20.8)	0.6219
KL-6 (U/ml), median (IQR)	342 (256–517)	424 (341–535)	415 (290–636)	336.5 (237–393)	0.1526
MMP-9 (ng/ml), median (IQR)	150 (93–209)	208 (90–283)	242 (163–374)	194 (102–229)	0.0246
PDGF-AA (pg/ml), median (IQR)	1,020 (768–1,697)	889 (647–1,236)	985.5 (702–1,497)	433.5 (282.8–665.8)	<0.0001
PIGF (pg/ml), median (IQR)	3.8 (3.2–4.3)	4.5 (4.2–4.9)	3.9 (3–4.6)	2.4 (1.8–3.3)	<0.0001
Thrombospondin-2 (ng/ml), median (IQR)	27 (21–36)	31 (24–44)	24.5 (16–38)	18.5 (15–25)	0.0027
VEGF-R3 (pg/ml), median (IQR)	182 (116.3–227)	165.1 (61.8–193.1)	168.9 (109.2–204.5)	86.94 (75.70–137.3)	0.0037
vWF (%), median (IQR)	143.9 (121.6–175.6)	157.3 (133.5–180.8)	97.15 (78–139.1)	124.2 (96.75–149.8)	0.0030

CA-125, cancer antigen-125; FGF-acidic, fibroblast growth factor-acidic; KL-6, Krebs von den Lungen 6; LAM, lymphangioleiomyomatosis; LT, lung transplantation; MMP, matrix metalloproteinases; OCLD, other cystic lung diseases; PDGF-AA, platelet derived growth factor-AA protein, PIGF, phosphatidylinositol-glycan biosynthesis class F protein; TSC, tuberous sclerosis complex; VEGF-D, vascular endothelial growth factor-D; vWF, von Willebrand factor.

**FIGURE 4:**
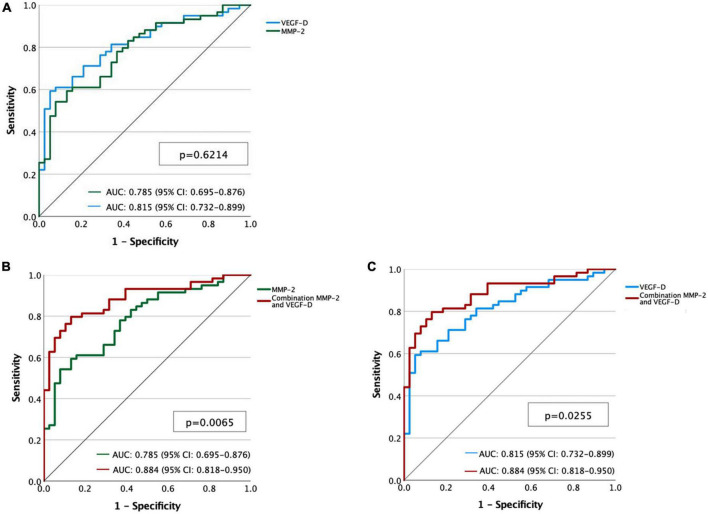
**(A)** Receiver operating characteristic (ROC) curves for metalloproteinase-2 (MMP-2) (green line) and vascular endothelial growth factor-D (VEGF-D) (blue line). **(B)** ROC curves of MMP-2 (green line) and the combination of MMP-2 and VEGF-D (red line). **(C)** ROC curves of VEGF-D (blue line) and the combination of MMP-2 and VEGF-D (red line).

The area under ROC curve (AUC) of VEGF-D to predict LAM diagnosis, compared with OCLD and healthy volunteers, was 0.815 [95% confidence interval (CI): 0.732–0.899, *p* < 0.0001]. A VEGF-D cut-off level of 578 pg/ml showed a sensitivity and specificity of 59 and 95%, respectively, whereas a cut-off value of 800 pg/ml had a sensitivity of 36 and a specificity of 97%, respectively. The AUC of MMP-2 was 0.785 (95% CI: 0.695–0.876, *p* < 0.0001). A cut-off level of 560 pg/ml showed a sensitivity of 54% for LAM diagnosis and a specificity of 92%. Specifically, a cut-off value of 824 pg/ml had a specificity of 100% to differentiate LAM from OCLD patients: 15 (25%) LAM patients fulfilled this criterion, 5 of them received a LT and 11 were under sirolimus treatment. A composite marker was therefore constructed combining MMP-2 and VEGF-D (score = −6.36 + 0.0059 × VEGF-D + 0.0069 × MMP-2). The AUC for this composite score was 0.88 (95% CI 0.82–0.95) (*p* < 0.05 compared to either VEGF-D or MMP-2 alone), and the best cut-off for the composite score was 0.61, resulting in a sensitivity/specificity of 79/86% (Figure 4).

We did not find any correlation between either MMP-2 or VEGF-D values and age, estradiol, luteinizing hormone, follitropin, progesterone, or prolactin levels. Nor was there any correlation between the two biomarkers. MMP-2 and VEGF-D were not associated with chylothorax or angiomyolipomas or with recurrencies; VEGF-D correlated with lymphatic involvement, but MMP-2 did not.

There was a nearly significant positive correlation between the levels of serum MMP-2 and the severity of the cystic lung involvement by CT: median MMP-2 was 458 (305–536) ng/ml in the mild disease group, 500 (348–783) ng/ml in the moderate disease group, and 551 (456–782) ng/ml in the severe disease group (*p* = 0.087). On the other hand, levels of serum VEGF-D were not correlated: median values were 574 (423–919), 503 (405–646), and 544 (444–816) pg/ml, respectively, for the three groups (*p* = 0.638).

**FIGURE 5:**
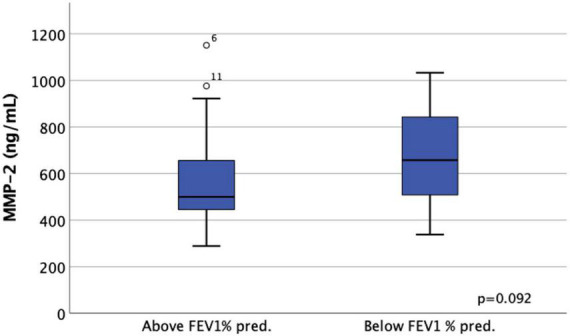
Classification of lymphangioleiomyomatosis (LAM) patients based on median FEV1% pred. higher or lower than 73.5%.

We observed a tendency toward an association between higher levels of serum MMP-2 and worse lung function parameters in LAM patients who had not undergone LT. In patients with FEV1% pred. below the median, mean MMP-2 was 674 ± 203 ng/ml, while in those above median FEV1% pred. it was 562 ± 226 ng/ml (*p* = 0.092), (Figure 5). VEGF-D did not correlate with lung function parameters.

## 4. Discussion

Finding new diagnostic biomarkers for LAM is a pressing issue. Our results show that serum levels of MMP-2 are significantly higher in patients with LAM compared to patients with OCLD and healthy volunteers. A composite score combining VEGF-D and MMP-2 can be used as a diagnostic biomarker for LAM and can increase the accuracy of the diagnosis.

Associated with lymphangiogenesis and lung remodeling, MMPs are components of the extracellular matrix that promote collagen and elastin degradation (13). MMPs are overexpressed in a number of chronic pulmonary diseases, including idiopathic pulmonary fibrosis (25, 26), asthma (27), and emphysema (28), as well as tumor proliferation (29). Hayashi et al. (30) demonstrated increased activity of MMPs, particularly MMP-2, which might be responsible for the cystic destruction of the lung parenchyma in LAM. Furthermore, these authors observed that the immunohistochemical heterogeneity of LAM cells was associated with different patterns; for example, larger LAM cells stained more strongly for MMP-2, MMP-9, and Human Melanoma Black-45 monoclonal antibody. According to other authors, the disproportion between MMPs and their tissue inhibitors may also lead to the cystic destruction of lung parenchyma (31).

Few studies have been published on MMPs and LAM. Chang et al. (17) found significantly higher MMP-2 serum levels among LAM patients, but they only compared them with healthy volunteers and excluded patients taking sirolimus and LT. Those authors hypothesized that MMP-2 may decrease in advanced disease, in view of an association they found between MMP-2 levels and better lung function. In contrast, we found that MMP-2 was associated with poorer lung function. Considering that a higher number of cysts, and therefore more lung destruction, impairs lung function, an increase in MMP-2 seems plausible in patients with poorer outcomes. In fact, we found a trend for an association between serum MMP-2 levels and the degree of lung involvement by CT.

A recent study explored the role of MMP-2 and MMP-7 as biomarkers for S-LAM and TSC-LAM in comparison to healthy controls (16). In both S-LAM and TSC-LAM patients, serum MMP-2 levels were higher than in controls, while MMP-seven levels were higher in TSC-LAM patients than in controls and S-LAM patients. However, higher levels of those biomarkers did not appear to correlate with worse lung function parameters. In the same study (16). MMP-2 did not have the same ability to diagnose LAM disease as VEGF-D, in agreement with our data; however, in that study the authors did not attempt to combine biomarkers in order to increase diagnostic accuracy. A Japanese study used semi-quantitative analyses to assess serum activity of MMP-9 and MMP-2 in LAM patients and compared them with healthy females (15). MMP-9 serum activity was significantly higher than in controls, but MMP-2 activity did not differ between groups. Due to the semi-quantitative nature of the method used for the analysis, the results of that study may differ from ours.

In addition, some authors have investigated the role of doxycycline, an MMP inhibitor, on LAM progression. Pimenta et al. (32) observed a decrease in MMP-2 levels after doxycycline treatment but this was not associated with functional improvement. On the other hand, Chang et al. (33) did not find differences in MMP-two levels after 2 years of doxycycline. Altogether, further studies should clarify the prognostic role of MMP-2 in LAM. We found VEGF-D levels to be significantly higher in LAM patients, with similar sensitivity and specificity values to those recorded in previous studies (6, 34). Young et al. (35) discovered significant correlations between measurements of VEGF-D at LAM diagnosis and lung function parameters. In contrast, we did not detect any association between lung function and serum VEGF-D levels, but our measurements were not made at baseline. Along the same lines, Young et al. (6) did not find any correlation in their cross-sectional study. Even though we observed no differences in VEGF-D levels between patients who received sirolimus and those who did not, this finding could be explained by the fact that patients not receiving sirolimus might have milder disease. In addition, the levels of pre-treatment VEGF-D are not available due to the design of the study. According to Taveira-DaSilva et al. (11) pre-sirolimus VEGF-D decreased especially in patients with lymphatic involvement, but they were unable to demonstrate an association between VEGF-D decline and lung function changes following sirolimus treatment. As a biomarker for monitoring the progression of LAM disease, VEGF-D has several limitations, a circumstance that highlights the importance of further research in this area.

Another relevant finding from our study is that VEGF-D remained high in LAM patients after LT. This result supports the hypothesis that LAM cells do not originate exclusively in the lungs, and that they may also proliferate in other tissues (36). Recently, Guo et al. (37) identified the presence of LAM cells in the uterus through single-cell RNA sequencing. To our knowledge, ours is the first study to measure VEGF-D after LT in LAM patients.

The main limitation of this study is its cross-sectional design. Also, the sample size was relatively small but this was due to the rarity of the disease and is a drawback for most LAM studies. The strengths of the study include the homogeneity of the clinical care and criteria, as all patients were treated by the same team.

In conclusion, a composite two-biomarker assay combining MMP-2 and VEGF-D enhances diagnostic accuracy. However, further longitudinal studies with larger patient populations are needed to confirm the usefulness of MMP-2 in clinical practice. The study also showed that serum levels of VEGF-D remained elevated in LAM patients even after LT.

## Data availability statement

The raw data supporting the conclusions of this article will be made available by the authors, without undue reservation.

## Ethics statement

The studies involving human participants were reviewed and approved by the Ethics Committee of the Vall d’Hebron University Hospital. The patients/participants provided their written informed consent to participate in this study.

## Author contributions

ER-L was responsible for collecting blood samples, data analysis, statistics, and writing of the manuscript. VR and AM-V were responsible for sample analysis and critically reviewed the manuscript. ML-M, CBe, MB-P, MA-P, MZ-O, VR, CBr, and AR critically reviewed the manuscript. SG-O and BS-G were responsible for study design and critical review. All authors contributed to the article and approved the submitted version.
