# Supplementary material for: Lymphangioleiomyomatosis: Searching for potential biomarkers
Source: Front Med (Lausanne). 2023 Feb 2;10:1079317. doi: 10.3389/fmed.2023.1079317 (PMC9931739; doi:10.3389/fmed.2023.1079317)
Supplement: Supplementary file 1 [file Data_Sheet_1.docx]

Supplementary Material

## Supplementary Table and Figures

| Treatments, n (%) | TSC-LAM (n=8) | S-LAM (n=36) | p-value |
| --- | --- | --- | --- |
| Sirolimus | 6 (75) | 27 (75) | 1 |
| Statins | 4 (50) | 12 (33.34) | 0.43 |
| AAR II | 0 (0) | 3 (8.34) | 1 |
| ACEI | 1 (12.5) | 4 (11.11) | 1 |
| Bronchodilator | 2 (25) | 13 (36.11) | 0.7 |
| Corticosteroid inhibitor | 1 (12.5) | 9 (25) | 0.66 |
| Antidepressant | 2 (25) | 11 (30.56) | 1 |
| Beta blockers | 2 (25) | 2 (5.56) | 0.15 |
| Hypoglycemiant agents | 1 (12.5) | 3 (8.34) | 0.57 |
| Hormonal treatment | 0 (0) | 3 (8.34) | 1 |
| Anticoagulants | 0 (0) | 2 (5.56) | 1 |
| Antiplatelet agents | 0 (0) | 3 (8.34) | 1 |

**Supplementary Table 1.** Comparison between TSC-LAM and S-LAM regarding other treatments


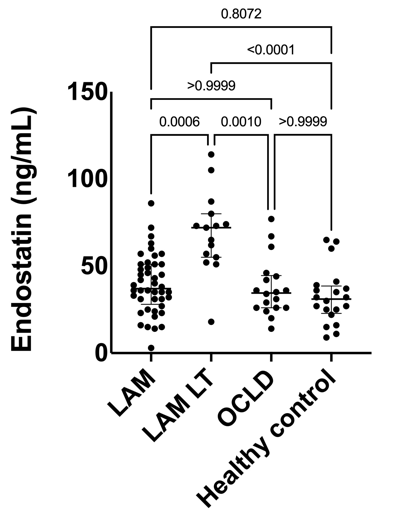

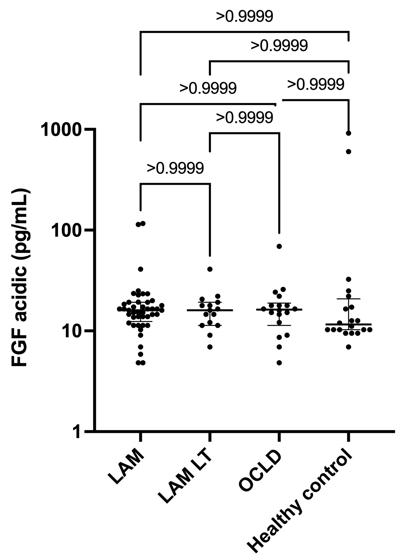

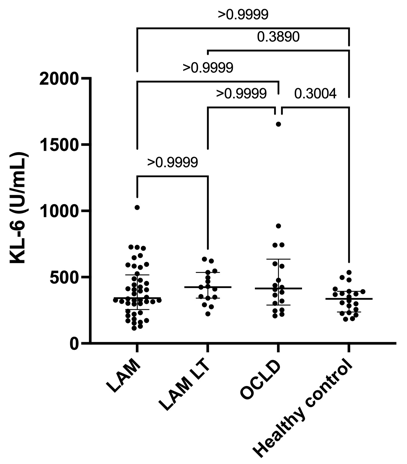

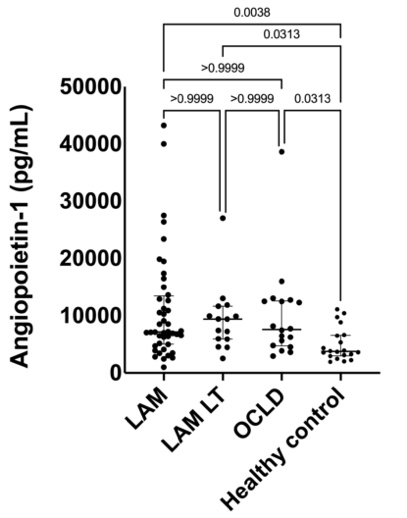

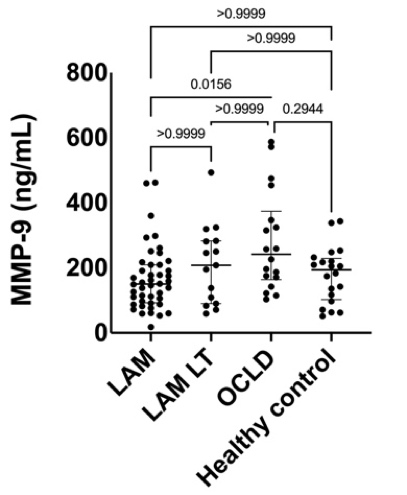

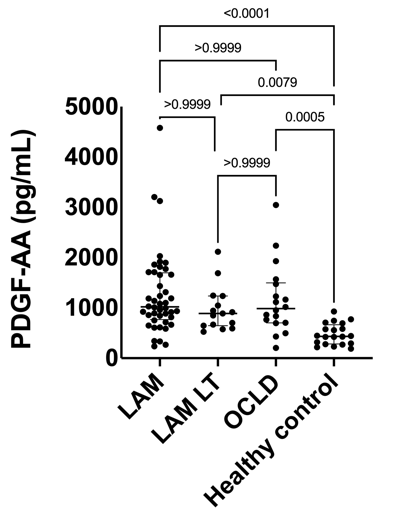

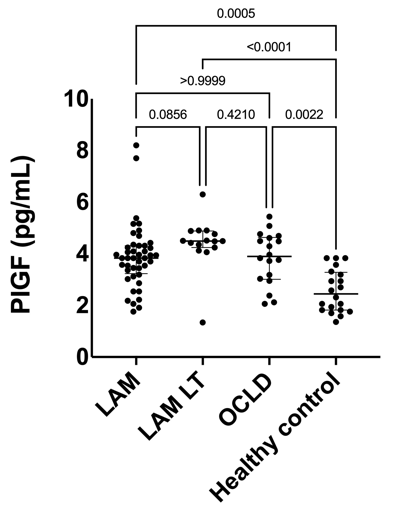

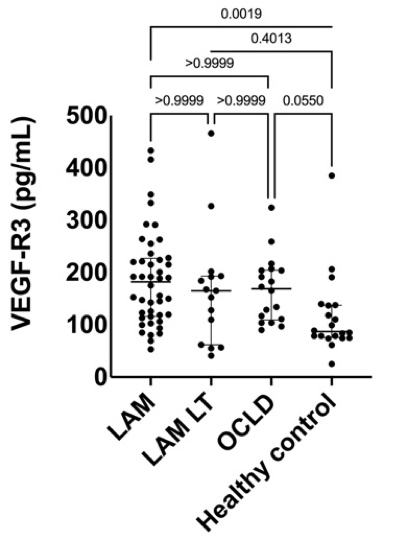

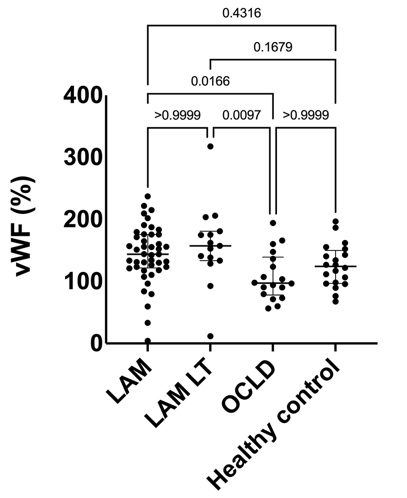

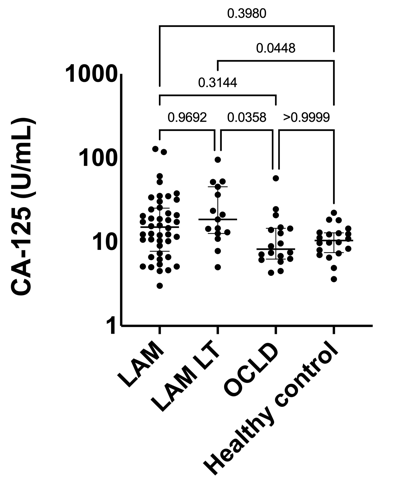

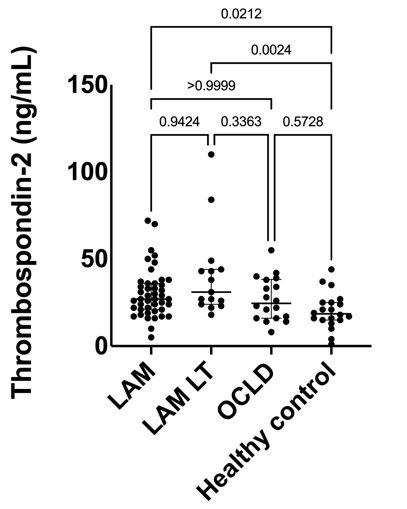


**Supplementary Figure S1**. Comparison of angiopoietin-1, CA-125, endostatin, FGF acidic, KL-6, MMP-9, PDGF-AA, PIGF, thrombospondin-2 VEGF-R3 and vWF between LAM patients, LT patients, other cystic lung diseases and healthy controls. Solid lines indicate median and interquartile range.


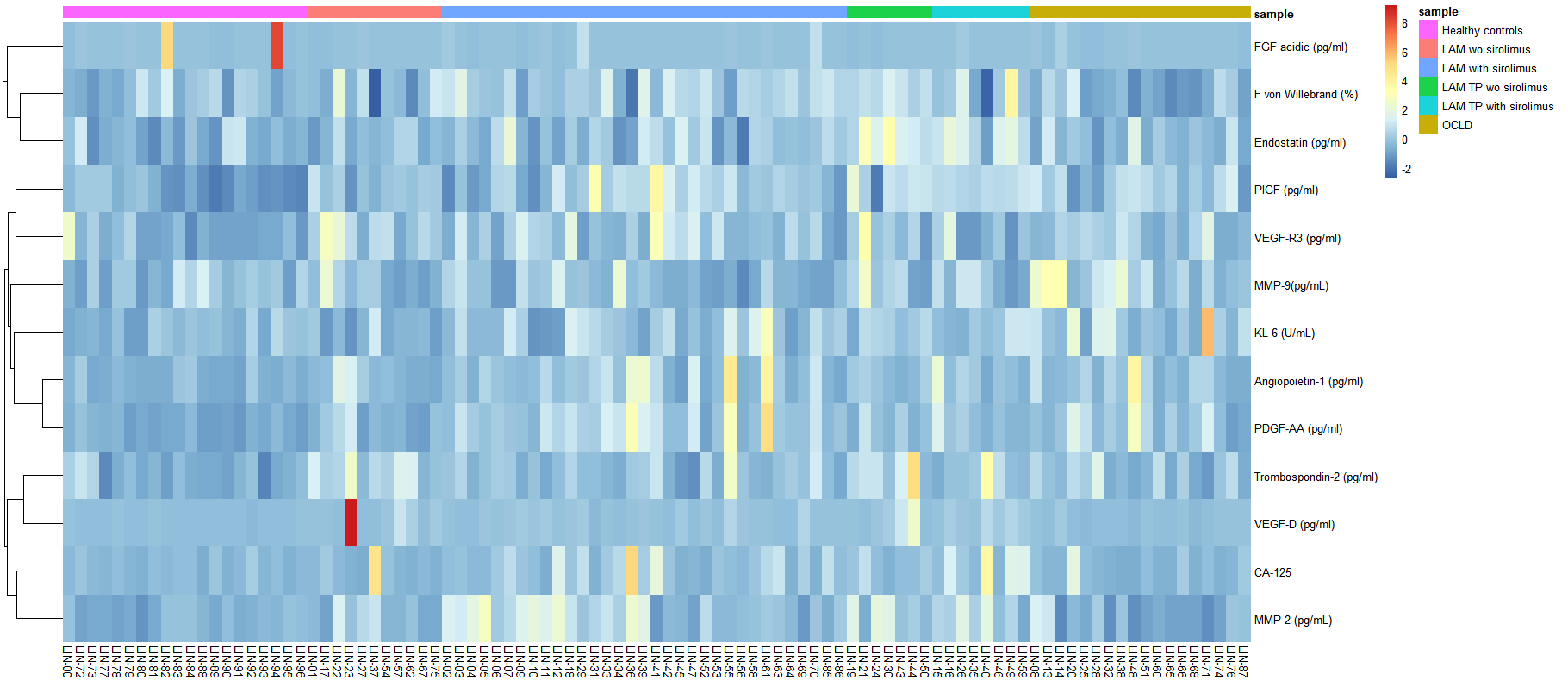

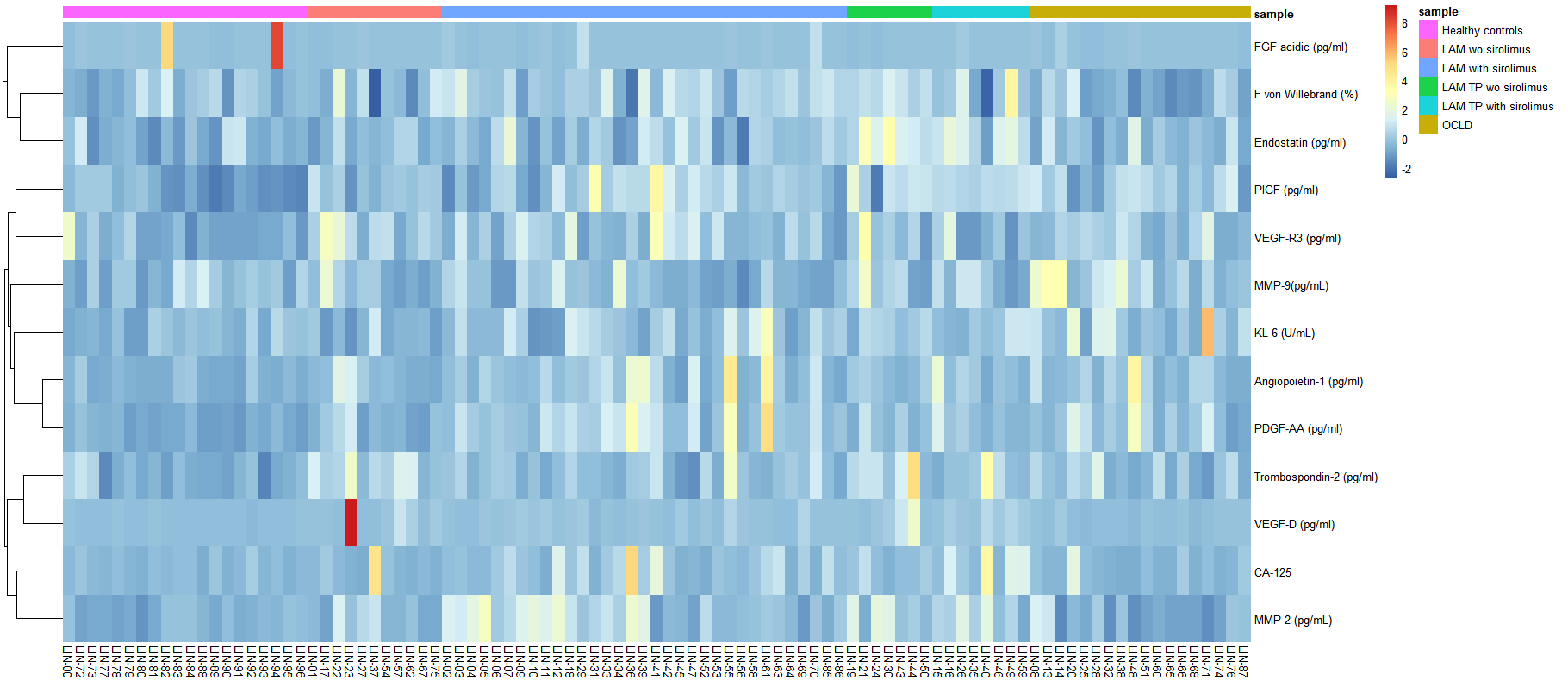

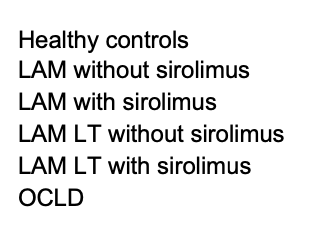


**Supplementary Figure S2**. Heatmap displaying grouped results. Each column represents a subject (grouped according to whether they are healthy controls, LAM patients who did not receive sirolimus treatment, LAM patients who received sirolimus, LT patients who did not receive sirolimus, LT patients who received sirolimus treatment, OCL) and each row represents a biomarker.
